# Charting the course of renal cryoinjury

**DOI:** 10.14814/phy2.12357

**Published:** 2015-04-20

**Authors:** Wasan Abdulmahdi, Joseph Zullo, Lauren Nesi, Michael S Goligorksy, Brian B Ratliff

**Affiliations:** 1 Department of Medicine, Renal Research Institute, New York Medical CollegeValhalla, New York, USA; 2 Department of Pathology, Renal Research Institute, New York Medical CollegeValhalla, New York, USA; 3 Department of Pharmacology, Renal Research Institute, New York Medical CollegeValhalla, New York, USA; 4 Department of Physiology, Renal Research Institute, New York Medical CollegeValhalla, New York, USA

**Keywords:** Apoptosis proliferation, cryoinjury, kidney injury model, renal blood perfusion

## Abstract

We sought to characterize a minor renal cryoinjury that allows investigation into renal damage processes and subsequent endogenous repair mechanisms. To achieve this, we induced a small cryoinjury to mice, in which the transient superficial application of a liquid nitrogen-cooled cryoprobe to the exposed kidney induces a localized lesion that did not impair renal function. The resulting cryoinjury was examined by immunohistochemistry and Laser-Doppler flowmetry. Within hours of cryoinjury induction, tubular and vascular necrotic damage was observed, while blood flow in the directly injured area was reduced by 65%. The injured area demonstrated a peak in tubular and perivascular cell proliferation at 4 days postinjury, while apoptosis and fibrosis peaked at day 7. Infiltration of macrophages into the injury was first observed at day 4, and peaked at day 7. Vascular density in the direct injured area was lowest at day 7. As compared to the direct injured area, the (peripheral) penumbral region surrounding the directly injured area demonstrated enhanced cellular proliferation (2.5–6-fold greater), vascular density (1.6–2.9 fold greater) and blood perfusion (twofold greater). After 4 weeks, the area of damage was reduced by 73%, fibrosis decreased by 50% and blood flow in the direct injured area was reestablished by 63% with almost complete perfusion restoration in the injury's penumbral region. In conclusion, kidney cryoinjury provides a flexible facile model for the study of renal damage and associated endogenous repair processes.

## Introduction

Despite the fact that regeneration after tissue injury can occur in amphibians and zebra fish (Poss et al. 2002; Tanaka 2003; Laube et al. 2006; Stoick-Cooper et al. 2007; Chablais et al. 2011; Schnabel et al. 2011; Chablais and Jazwinska 2012), postinjury tissue regeneration with restoration of the normal architecture in adult mammals is highly limited (Laflamme and Murry 2005; Murry et al. 2006; Rubart and Field 2006). Nonetheless, it has been discovered that complete regeneration has the potential to occur in adult mice; when the terminal digit phalanx is amputated in a mouse, it is able to regrow (Schotte and Liversage 1959; Borgens 1982; Neufeld and Zhao 1993; Reginelli et al. 1995; Han et al. 2008; Rinkevich et al. 2011), similar to what occurs in young human children (Illingworth 1974; Rosenthal et al. 1979; Vidal and Dickson 1993). In addition, the liver of adult humans also has the ability to regenerate and retains its original size and function after partial hepatectomy (Lin and Chen 1965). In contrast, while mammalian kidneys can repair minor acute damage (Poulsom et al. 2001, 2003; Bonventre 2003; Kale et al. 2003; Lin et al. 2003; Maeshima et al. 2003; Morigi et al. 2004), they are unable to fully regenerate after a substantial injury, as is the liver. In recent years, intense effort has been placed on investigations aimed at understanding and enhancing the kidney's ability to regenerate after substantial damage. Studies utilizing models of acute renal insults, such as ischemia and toxin-induced nephropathy, have provided insight that nephron repair occurs primarily by proliferation and migration of surviving cells adjacent to specific sites of damage (Berger et al. 2014; Kusaba et al. 2014; Rinkevich et al. 2014). However, the underlying mechanisms responsible for these processes remain unclear.

Cryoinjury has been previously reported on in heart models and has allowed the investigation into cardiac repair mechanisms (Lefer et al. 1986; Ciulla et al. 2004; van den Bos et al. 2005; van Amerongen et al. 2008; Chablais et al. 2011; Gonzalez-Rosa et al. 2011; Schnabel et al. 2011; Chablais and Jazwinska 2012; Gonzalez-Rosa and Mercader 2012). The cryomodel has successfully competed with another more traditional model, ligation of the coronary artery, in multiple studies of myocardial injury. In the field of studies into kidney injury, an analogous cryomodel has not previously been investigated. However, the damage sustained and subsequent repair processes that occur after introduction of a localized kidney cryoinjury are more amenable to investigations than a global kidney injury, by analogy with the terminal digital amputation and regeneration observed in adult mice (Rinkevich et al. 2011). Therefore, we sought to optimize the existing cryoinjury model for the use in the kidney and characterize the dynamics of pathological and regenerative processes taking place after a small localized cryoinjury induced to a murine kidney by briefly applying a liquid nitrogen cooled cryoprobe to the surface of an exposed mouse kidney. While the introduced cryoinjury was minor in size (1.5 mm in diameter) and did not impair renal function, it did induce substantial localized vascular/tubular damage. Within 1 month of initial insult, the cryo-induced lesion demonstrated significant repair. Interestingly, during the reparative phase, the cryo-injured kidney displayed three well-demarcated zones: (1) a direct injury with necrotic cells, (2) a penumbra, region around the peripheral edge of the necrotic area and (3) a visibly unperturbed peripheral zone of the kidney. The penumbral region was characterized by enhanced cellular proliferation, vascular density and blood perfusion. Here, we describe the optimal protocol for technical procedures and chart the course of cryoinjury and its repair in the kidney. The establishment of a cryo-induced kidney injury model has potential to facilitate further investigations into intrinsic regenerative process that occurs in the mammalian kidney, particularly in regard to the reparative dynamics associated with the penumbral injured region. Insight gained into the repair mechanisms through the use of this model may be used to manipulate and maximize the renal tissue regenerative capacity in hopes of improving therapeutic strategies for treatment of acute and chronic kidney injury.

## Materials and Methods

### Animal studies

The animal study protocol was in accordance with the National Institutes of Health (NIH) Guide for the Care and Use of Laboratory Animals and approved by the Institutional Animal Care and Use Committee of New York Medical College. Adult male 10 week old FVB/NJ mice were purchased from the Jackson Laboratory (Bar Harbor, ME) and used in experiments. All animals were separately caged with an (12:12) hour light–dark cycle and had free access to water and chow throughout the study.

### Surgical procedures

**Figure 1:**
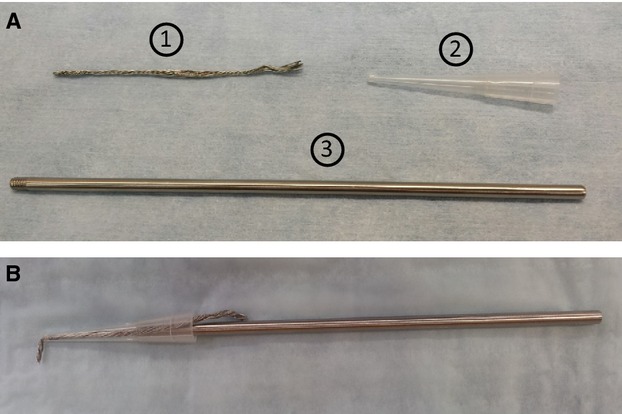
Design and construction of the cryoprobe. (A) The cryoprobe was constructed out of (1) brass braided nickel-coated 12 strand flexible wire, (2) a 200 *μ*L pipet tip, and (3) a stainless steel threaded rod that was 3 mm wide and 13 cm long. For construction, the braided wire was pulled through the fine point of the 200 *μ*L pipette. The wire-pipet piece was then screwed onto the threaded end of the stainless steel handle to obtain the final cryoprobe (B). The length of wire that was pulled through the pipette was bent at a 90° angle and was subject to liquid nitrogen cooling for subsequent application to the kidney for induction of cryoinjury. The length of the wire pulled through the pipet tip can be altered to induce a cryoinjury of variable size. In our experiments, the length of wire that was pulled through was 1.5 mm. The 200 *μ*L pipet tip serves to hold the wire in place, for attachment to the steel rod, and also serves as a handle tip to protect the user after the cryoprobe has been cooled by liquid nitrogen. The design of the cryoprobe was based on a previous design by Gonzalez-Rosa and Mercader with slight modifications (Gonzalez-Rosa and Mercader 2012). Description and images of our modified probe design are presented here with permission from Gonzalez-Rosa and Mercader.

FVB/NJ mice were anesthetized using isoflurane, USP (5% for induction and 1–3% for maintenance) (Butler Schein, Dublin, OH). Mice were placed on a heated surgical pad and body temperature was maintained at 37°C. The kidneys were exposed by a 1-cm midlaparotomy incision. Cryoinjury was induced bilaterally using a liquid nitrogen cooled cryoprobe that we constructed in-house (Fig.1) following a design previously described (Gonzalez-Rosa and Mercader 2012). Injury size was 1.5 mm in diameter and was induced superficially on the lower pole of kidneys by applying the cryoprobe to the kidney surface at constant pressure for 20 sec. The kidney capsule was not removed prior to or after application of the cryoprobe to the surface of the kidney. After bilateral cryoinjury induction, we immediately treated one of the bilaterally injured kidneys with Leukemia Inhibitory Factor (LIF) by local injection of LIF (0.2 *μ*g diluted in 10 *μ*L of normal saline vehicle) directly at the site of cryoinjury (injection was made superficially just beneath the kidney capsule at the site of injury). This treatment was designed to examine the therapeutic efficacy of LIF in accelerating healing of the cryoinjury. However, the treatment was noneffective in accelerating healing of the bilateral kidney, thus we report only on the contralateral cryo-injured kidney that did not receive any (LIF) treatment. Afterwards, incisions were sutured, mice were treated with buprenorphine (0.5–1 mg/kg) (Reckitt Benckiser Healthcare Ltd., Richmond, VA) intraperitoneally and monitored for recovery during the following 24 h. Incisions sites were treated with antibiotic ointment.

We standardized the cryoprobe application protocol in order to induce a consistent and reproducible cryoinjury to kidneys. First, the cryoinjury was induced at the same site, the lower pole of the kidney. We observed that slight locality variations of injury induction to the cortical tissue of the lower pole of the kidney were negligible, as based on similarities in lesion size, cell death and proliferation, fibrosis, blood perfusion, and rate of repair. However, we avoided inducing injury in the vicinity of the renal pedicle or close to the adrenal gland. Secondly, in order to apply the probe at a consistently cooled temperature, we standardized the length of time that was allotted after removal of the probe from its precooling in liquid nitrogen until its application to the kidney surface. We standardized this time to 5 sec. If the cryoprobe could not be accurately applied to kidneys within this time frame, then the cryoprobe was recooled by immersion in liquid nitrogen. We also sought to avoid movement and pressure irregularities during probe application to the kidney. To overcome this concern, we attached the cryoprobe to a mechanical micromanipulator and applied the probe to the kidney surface via the micromanipulator. The micromanipulator was positioned prior to probe cooling and attachment. Once precooling was complete, the cryoprobe was inserted into the manipulator and immediately applied to the kidney. Application of the probe to the kidney surface was precisely timed and promptly removed upon conclusion of the strictly timed interval. Collectively, these standardized procedures effectively removed much of the variations initially observed during application of the cryoprobe.

At varying time points after surgery (1, 2, 4, 7, 14, and 28 days), renal blood flow was examined in mice prior to sacrifice and sample collection. Mice were anesthetized with ketamine (100 mg/kg) (Henry Schein, Dublin, OH) and xylazine (10 mg/kg) (Lloyd Laboratories, Shenandoah, IA) and renal blood perfusion in the site of injury was measured by Laser-Doppler flowmetry (as described in detail here later). Prior to laparotomy, a laser-Doppler Flowmetry scanner was used to obtain pseudocolored scan images of renal blood flow in live anesthetized mice. Subsequently, Laser-Doppler flowmetry probes were used after laparotomy (to expose kidneys) to obtain specific renal blood perfusion values in live anesthetized mice. After Laser-Doppler renal blood flow analysis, mice were euthanized by cardiac puncture and cervical dislocation. Upon euthanasia, normal saline was perfused through the left ventricle, and kidneys were harvested. After perfusion, each kidney was imaged using a pin microscope (Hammacher Schlemmer, Fairfield, OH) with 4× magnification to obtain images of the specific cryoinjured area on the kidney surface. After excision, kidneys were fixed in 4% paraformaldehyde, dehydrated with 30% sucrose or 70% ethanol, embedded in OCT (Tissue Tek, Torrance, CA) or paraffin, and stored at −80°C (OCT samples) until immunofluorescence analysis (Park et al. 2010a).

### Histologic analysis

For hematoxylin and eosin (H&E) and Masson's Trichrome staining, kidney sections fixed in 4% paraformaldehyde (PFA) (Electron Microscopy Sciences, Hatfield, PA) were embedded in paraffin. Samples were sectioned at 4 *μ*m in thickness and stained with H&E (Park et al. 2010b) or Masson's trichrome (Rabadi et al. 2012), as previously described. Stained kidney sections were imaged on a Nikon Eclipse TE 2000-U microscope (Morrell, Melville, NY) equipped with a SPOT Insight QE color camera (model 4.2). The acquisition software used was SPOT imaging software (version 4.0.1, Diagnostic Instruments, Sterling Heights, MI). H&E and Masson's trichrome staining was analyzed by ImageJ software (U.S. National Institutes of Health, Bethesda, MD). Masson's trichrome staining was evaluated by ImageJ's Integrated Density programming.

### Immunofluorescence analysis

Kidneys embedded in OCT were cryosectioned at a thickness of 20 *μ*m and placed on probe-on plus slides (Fisher Scientific, Pittsburgh, PA). Sections were permeabilized with Triton X-100 in 0.2% PBS/BSA and blocked with 0.2% PBS/BSA for 1 h. Sections were incubated with primary antibodies overnight at 4°C. Secondary antibodies were applied the following day for 1 h. Following washes in PBS, nuclei of cells were stained with (10 mg/mL) 4′, 6-diamidino-2-2 phenylindole dihydrochloride (DAPI) (Sigma-Aldrich, St. Louis, MO). Sections were examined for immunofluorescence using the previously described microscope equipment and software. Quantification of Ki-67, TUNEL, F4/80 and CD11c was performed by counting the number of positive stained cells within the tubular and perivascular compartments within and around the cryoinjury, as observed in each captured 400× magnified high-powered field (HPF). The number of positive cells in each image was normalized per number of tubules observed in each HPF. The tubular and perivascular compartments were identified by tubular autofluorescence and DAPI staining. CD31 staining was quantified by placing a digital grid (containing 266 equally sized boxes) over each HPF image and evaluating the percentage of grid boxes that were positive for CD31 staining.

Primary antibodies used in this study were: monoclonal rat anti-mouse CD31 (BD Pharmingen, San Jose, CA) (dilution 1:250), polyclonal rabbit anti-mouse Ki-67 (Abcam, Cambridge, MA) (dilution 1:250), monoclonal rat anti-mouse F4/80 (eBioscience, San Diego, CA) (dilution 1:200) and monoclonal Armenian Hamster anti-mouse CD11c (dilution 1:100) (Abcam). Secondary antibodies used in this study were: AlexaFluor 488- or 594-conjugated goat anti-rat (dilution 1:500) (Life Technologies, Grand Island, NY), AlexaFluor 594-conjugated goat anti-rabbit (dilution 1:300) (Life Technologies), and AlexaFluor 488-conjugated goat anti-Armenian Hamster (1:1000) (Life Technologies).

### TUNEL staining

Sections were examined for apoptosis using the In Situ Cell Death Detection (TUNEL) TMR red assay kit (Roche, Indianapolis, IN). In brief, OCT kidney cryosections were permeabilized with 0.2% Triton PBS/BSA for 2–5 min, followed by incubation with the kit's TUNEL reaction mixture (containing nucleotides and terminal deoxynucleotidyl transferase) for 60 min at 37°C. Afterwards, total nuclei were stained with (10 mg/mL) DAPI. Positive controls were obtained by incubating sections with DNase I (1:100) (Qiagen, Valenica, CA) for 10 min at 15–25°C prior to labeling procedures. Negative controls were obtained by incubating sections with the labeling solution without terminal deoxynucleotidyl transferase. Staining was subsequently examined by immunofluorescence microscopy using excitation wavelength 520–560 nm and detection in the range of 570–620 nm.

### Laser-Doppler flowmetry

At varying time points after cryoinjury induction (1, 2, 4, 7, 14, 28 days), Laser-Doppler flowmetry (Perimed, Jarfalla, Sweden) was utilized to determine renal blood perfusion within and around the area of injury. Mice were anesthetized by intraperitoneal injection of ketamine (100 mg/kg) and xylazine (10 mg/kg). Prior to laparotomy, a laser-Doppler Flowmetry scanner was used to obtain pseudo colored scans of renal blood flow in live anesthetized mice. Subsequently, Laser-Doppler flowmetry probes were used after laparotomy (to expose kidneys) to obtain specific renal blood perfusion values in live anesthetized mice. For laparotomy, a mid-line incision was applied to expose both kidneys, as previously described here. Renal blood flow measurements and images were acquired using the Laser-Doppler flowmetry probes (Periflux System 5000) and the Perimed Periscan PIM II Laser-Doppler perfusion scanner imager, respectively.

### Sampling size and statistical analysis

Regarding experimental sample size, three separate animals that all received kidney cryoprobe injury; *n* = 3, were used for each time point in H&E and Masson's Trichrome analyses. For immunofluorescence analysis, a larger sample size (five replicates; *n* = 5 animals) was used for each time point examined. For analysis of renal blood perfusion by Laser-Doppler flowmetry, six replicates were used (*n* = 6 animals) for each time point examined. Each cryo-injured animal was used exclusively for only one time point. Animals were randomly assigned to specific time points after induction of cryoinjury in order to ensure complete randomization and to avoid introduction of any systemic bias into experiments and statistical analysis. No animals died or were discarded during or after induction of cryoprobe injury due to complications. The absence of death and complications, in addition to the consistency in the time course of damage and repair events, further underscores the benefits of the cryoinjury model.

Data are presented as means ± SEM. Data presented as a means of a limited number of replicates (*n *≤ 6) was analyzed using nonparametric methods. The Kruskal–Wallis test was used to compare three or more groups of nonparametric data, and Dunn's Multiple Comparison test or the Mann–Whitney *U* test was subsequently performed to identify significant differences by pairwise comparison of each group. Statistical analysis softwares used were GraphPad Prism version 4.00 for Windows (GraphPad Software, San Diego, CA) and NCSS9 (Kaysville, UT). Differences were considered significant at *P *≤ 0.05, unless otherwise denoted.

## Results

The cryoprobe used in experiments is illustrated in Figure1. We initially examined application of the cryoprobe to the kidney for variable durations. Application of the cryoprobe to the kidney for durations <20 sec did not induce a substantial visible lesion on the surface of the kidney. In contrast, longer application of the cryoprobe of 20 sec did induce a visible lesion on the kidney surface. Therefore, we examined the cryoinjury that was induced by cryoprobe application of 20 sec when the lesion first began to appear on the kidney surface. We did not extend the duration of cryoprobe application beyond 20 sec because we did not want to induce a wound that was so severe that potential repair of the damaged tissue would be impaired.

**Figure 2:**
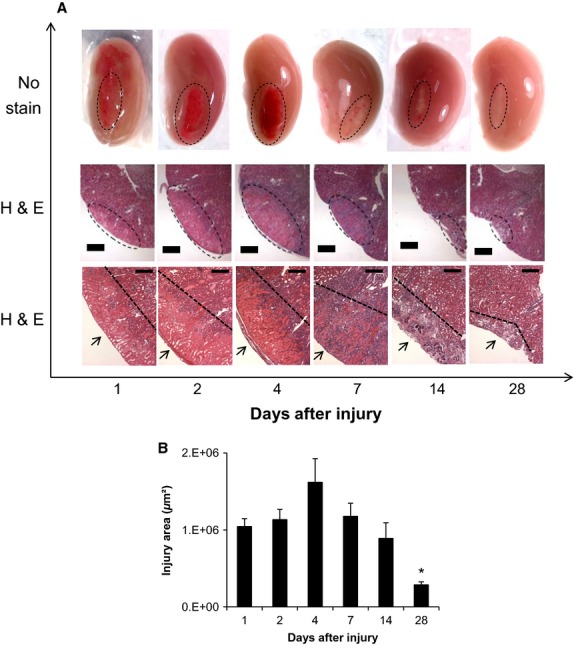
The time lapse course of kidney cryoinjury. (A) Top row: Illustration of time course differences in size and appearance of the cryoinjury on the kidney surface (magnification 10×). Images were acquired by pin microscope after saline perfusion. Saline perfusion prior to imaging removed red blood cells and further highlighted the area of cryoinjury. Middle row: Representative H&E images of the injured area (magnification 40×). The injured area is outlined by the dashed oval. Scale bar represents 400 *μ*m. Bottom row: Representative H&E images illustrate the injured area and the time course of leukocyte infiltration. The cryoinjury is indicated by the arrow while the dashed line outlines the injury. Scale bar represents 200 *μ*m. (B) Quantification of the injured area indicated the injury initially expanded in size, but later regressed 4 weeks after injury induction. *P < 0.05 day 28 versus day 4; *n* = 3 animals per time point.

**Figure 3:**
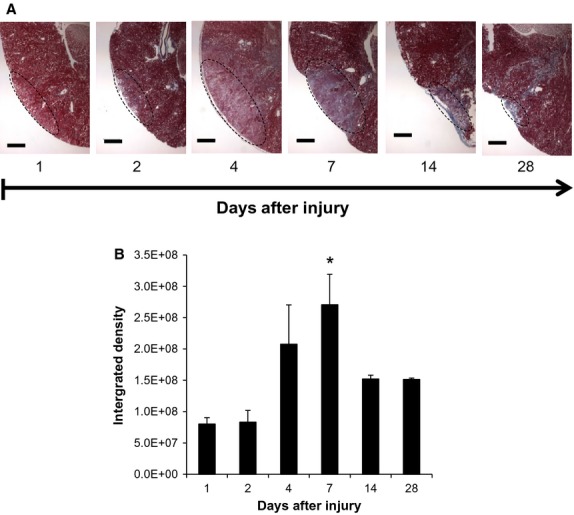
Fibrosis progression in the kidney after cryoinjury, as demonstrated by Masson's Trichrome. (A) Illustrative Trichrome images of fibrosis in the cryo-injured area (magnification 40×). The fibrotic area is identified by the dashed oval. (B) Trichrome staining and fibrosis in the kidneys was quantified by ImageJ and is represented as integrated density. Scale bar represents 400 *μ*m. *P < 0.05 versus day 1; *n* = 3 animals per time point.

Application of the cryoprobe for 20 sec to the surface of the surgically exposed kidneys (lower pole) induced a small localized wound (1.5 mm in diameter) that was characterized by swelling and redness (Fig.2A). On day 1, there was also sporadic redness on the kidney surface that extended outside of the direct cryo-injured area, which disappeared by day 2. The color eventually turned white in appearance in the following weeks. Renal function was not impaired in animals, as the cryoprobe injury was small in size. H&E staining of kidney cross-sections revealed leukocyte infiltration in and around the injury site occurring soon after injury induction with the maximal infiltration occurring between 4 and 14 days postinjury (Fig.2A). The cryolesion expanded in size by 55% within the first 4 days after initial cryoinjury, as indicated by necrosis and a lack of nuclear staining by H&E. After day 4, the size of the lesion on the kidney surface gradually declined in size through day 28, at which time the size of the injury was reduced by 73% (Fig.2B). Staining with Masson's Trichrome (Fig.3A) revealed fibrosis was increased at the site of the injury at day 4 postinjury, peaked at day 7 with an almost 2.5-fold increase, and then regressed by almost 50% at later time points (Fig.3B).

**Figure 4:**
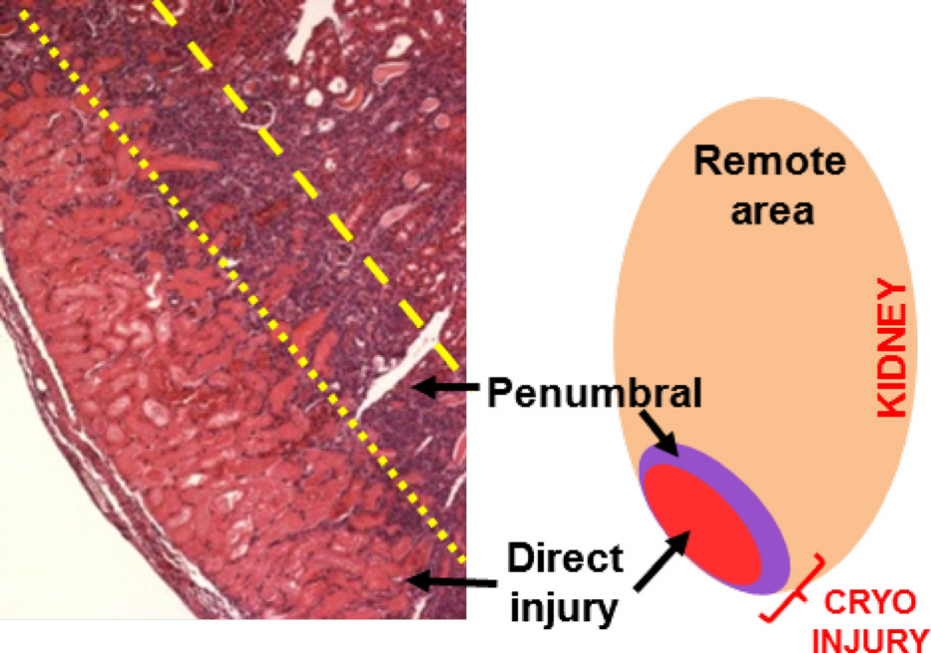
Illustration of the direct and penumbral injured areas. The H&E image illustrates and the schematic diagrams the direct and penumbral injured areas observed in the kidney after cryoinjury. The remote area of the kidney is also indicated in the schematic diagram. The direct injured area was the kidney tissue most severely damage due to direct cryoprobe exposure. The direct injured area reached a depth of approximately 400 *μ*m. Around the peripheral of the direct injured area was the penumbral injured area, which was a transition zone between severely injured tissue and the uninjured remote area. The penumbral injured area was approximately 200 *μ*m thick.

**Figure 5:**
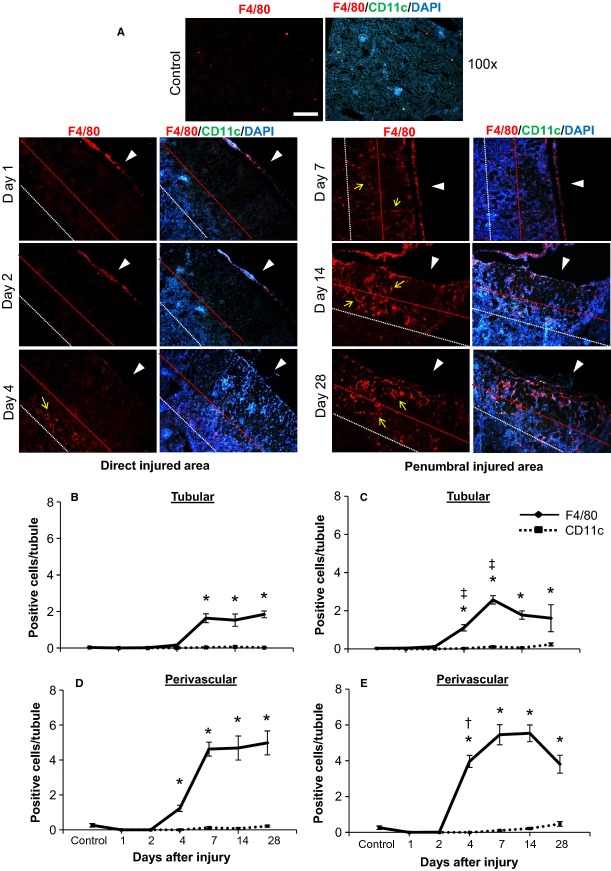
Quantification of macrophage infiltration in the kidney cryoinjury, as determined by positive F4/80 immunofluorescence staining. (A) Representative images include both the direct and penumbral cryo-injured areas at 1, 2, 4, 7, 14 and 28 days postinjury. The left panel illustrates F4/80 (red) staining alone with F4/80 positive cells indicated by yellow arrows. The right panel illustrates F4/80 (red), CD11c (which stains dendritic cells) (green) and DAPI (blue) merged images. White arrow heads indicate the direction in which the cryoprobe was applied to the kidney surface to induce injury. The outline of the direct injured area is indicated by the red dashed line, while the area between the red and white dashed lines is the peripheral penumbral injured area. F4/80 and CD11c positive cells in the direct (B,D) and penumbral (C,E) injured areas were quantified separately in the tubular (B,C) and perivascular compartments (D,E). F4/80 and CD11c positive cells were normalized relative to the total number of tubules observed in each image. *P < 0.05 versus control; ^†^*P *< 0.05 direct injured area (perivascular) versus penumbral injured area (perivascular) – same day; ^&ddagger;^*P *< 0.05 direct injured area (tubular) versus penumbral injured area (tubular) – same day; *n* = 5 animals per time point (except day 28 had an *n* = 4). All images are 100× magnification. Scale bars represent 200 *μ*m. Minor contrast and brightness adjustments were made to images to reduce background noise, but only after quantification of staining was completed.

In subsequent analysis using various immunofluorescence markers, we sought to examine if there were differences in repair dynamics between regions within the injury. Toward this end, we compared macrophage infiltration, cell proliferation, apoptosis, vascular density and blood flow between the severely injured area that was directly exposed to the cryoprobe and the peripheral moderately damaged tissue (termed the penumbral injured area) surrounding the direct injured area. We utilized DAPI fluorescence staining to identify the penumbral region in sectioned kidneys. While the direct injured area lacked DAPI staining, in contrast, the peripheral penumbral region demonstrated discontinuous DAPI staining in an approximately 200 *μ*m wide zone that surrounded the direct injured area. We used this patchy staining pattern of DAPI to identify the penumbral region. However, identification of the penumbral area remained relatively arbitrary. An illustration of the penumbral and direct injured areas is portrayed in Figure4, in which the day 4 H&E image indicates the two different regions of the cryoinjury, while the accompanying schematic further highlights their location and assigned zoning. An example of the differences in DAPI staining between the two regions is also represented in Figure5. The kidney capsule was not removed prior to cryoprobe application and was consequently also damaged during cryoinjury. After superficial cryoinjury, demarcation of the kidney capsule was not always possible and as a result capsular damage and repair is included in the general analysis of the direct injured area.

In Figure5, in addition to DAPI staining, macrophage infiltration was examined by F4/80 immunofluorescence staining. Macrophages play a critical role in tissue injury and repair (Wynn et al. 2013). After injury, circulating monocytes/macrophages enter damaged tissue, release cyto-/chemokines, clear cellular debris, and begin promoting revascularization and reepithelialization. In the cryoinjury, macrophages (as stained by F4/80) were first observed significantly infiltrating the perivascular and tubular compartments of the penumbral injured area at day 4, subsequently followed by infiltration of the direct injured area at day 7 (Fig.5). However, macrophage infiltration occurred more rapidly and was more robust (by upwards of 2.5-fold) in the perivascular compartment, as compared to the tubular compartment. F4/80 positive cells were confirmed to be macrophages and not dendritic cells by the lack of co-staining with the dendritic marker CD11c (Fig.5).

**Figure 6:**
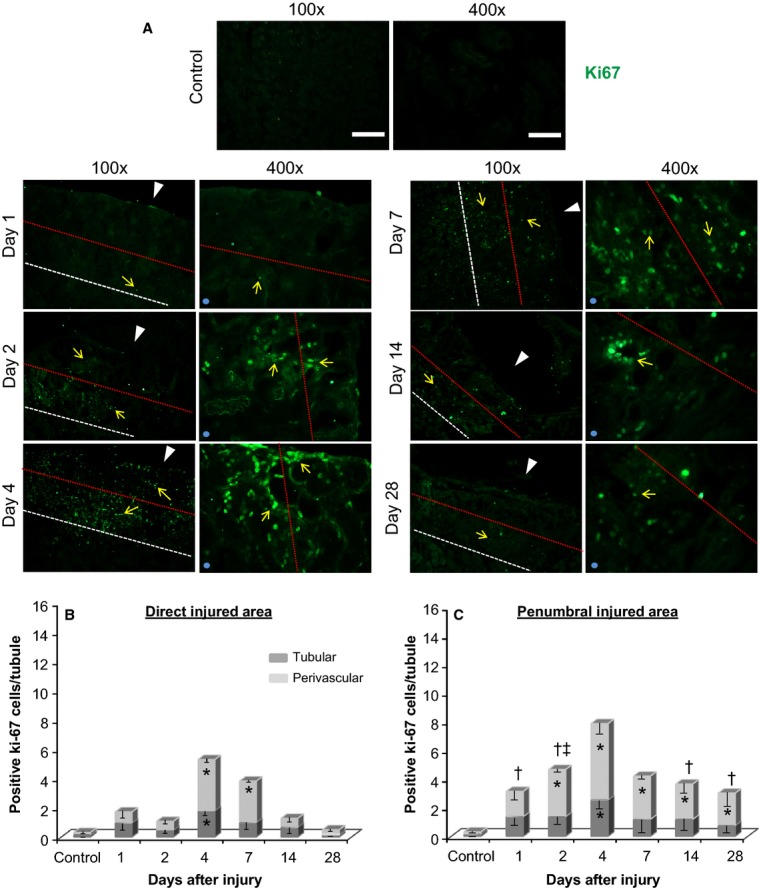
Cell proliferation (positive Ki-67 immunofluorescence staining) in the kidney cryoinjury at various time points after initial injury. (A) Representative images include both the direct and penumbral cryo-injured areas at 1, 2, 4, 7, 14 and 28 days postinjury. Left panel images were taken at 100× magnification and illustrate the direct and penumbral injured areas. Right panel images were taken at 400× magnification and illustrate the penumbral-direct injured area junction, as indicated by the red dashed line (the side that represents the penumbral injured area is indicated by the light blue sphere in the lower left corner of images). Positive Ki-67 staining is in green and is indicated by the yellow arrows. White arrow heads indicate the direction in which the cryoprobe was applied to the kidney surface to induce injury. The outline of the direct injured area is indicated by the red dashed line, while the area between the red and white dashed lines is the peripheral penumbral injured area. (B) Ki-67 positive cells in the direct (B) and penumbral (C) injured areas were quantified in the tubular and perivascular compartments. Ki-67 positive cells were normalized relative to the total number of tubules observed in each image. *P < 0.05 versus control; ^†^*P *< 0.05 direct injured area (perivascular) versus penumbral injured area (perivascular) – same day; ^&ddagger;^*P *< 0.05 direct injured area (tubular) versus penumbral injured area (tubular) – same day; *n* = 5 animals per time point (except day 28 had an *n* = 4). Scale bars represent 200 *μ*m in 100× magnification, and 50 *μ*m in 400× magnification. Minor contrast and brightness adjustments were made to images to reduce background noise, but only after quantification of staining was completed.

We next examined cellular proliferation in the cryo-injured area by staining for Ki-67 (Fig.6A), including quantification of proliferative differences in the tubular and perivascular compartments (Fig.6B and C). Cell proliferation (Ki-67 positive staining) was consistently more robust in the perivascular, compared to tubular, compartment. While both the direct and penumbral injured areas peaked in cell proliferation at 4 days postinjury, proliferation was 2.5–6 fold greater in the perivascular compartment of the penumbral region at earlier (days 1 and 2) and later (days 14 and 28) time points (Fig.6B and C), as compared to the direct injured area.

**Figure 7:**
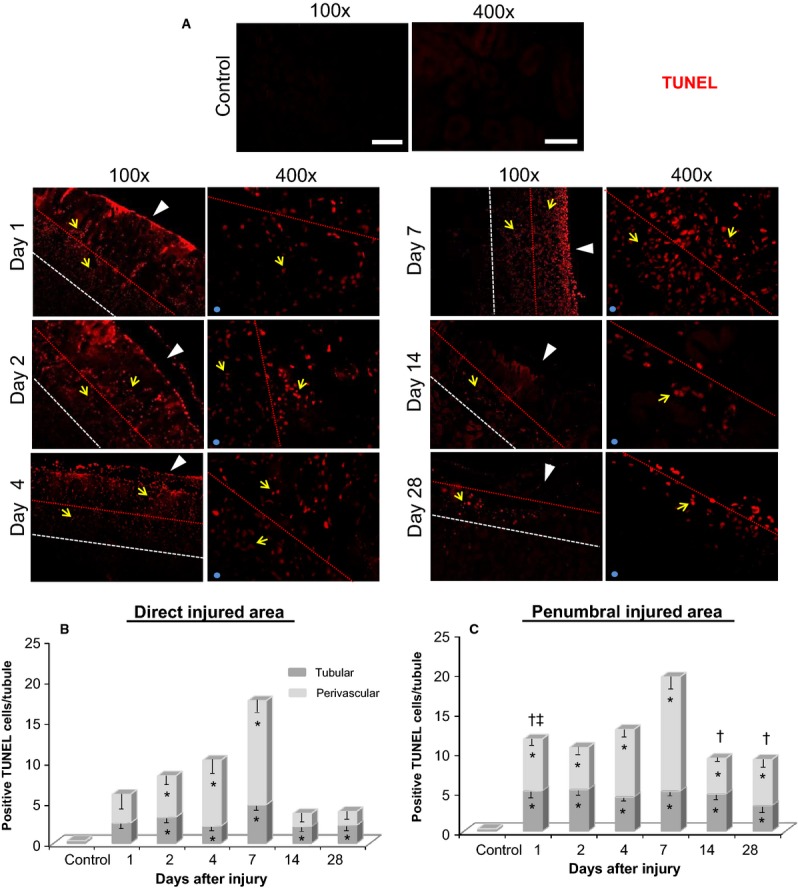
Apoptosis (positive TUNEL immunofluorescence staining) in the kidney cryoinjury at various time points after initial injury. (A) Representative images include both the direct and penumbral cryo-injured areas at 1, 2, 4, 7, 14 and 28 days postinjury. Left panel images were taken at 100× magnification and illustrate the direct and penumbral injured areas. Right panel images were taken at 400× magnification and illustrate the penumbral-direct injured area junction, as indicated by the red dashed line (the side that represents the penumbral injured area is indicated by the light blue sphere in the lower left corner of images). Positive TUNEL staining is in red and is indicated by the yellow arrows. White arrow heads indicate the direction in which the cryoprobe was applied to the kidney surface to induce injury. The outline of the direct injured area is indicated by the red dashed line, while the area between the red and white dashed lines is the peripheral penumbral injured area. (B) TUNEL positive cells in the direct (B) and penumbral (C) injured areas were quantified in the tubular and perivascular compartments. TUNEL positive cells were normalized relative to the total number of tubules observed in each image. *P < 0.05 versus control; ^†^*P *< 0.05 direct injured area (perivascular) versus penumbral injured area (perivascular) – same day; ^&ddagger;^*P *< 0.05 direct injured area (tubular) versus penumbral injured area (tubular) – same day; *n* = 5 animals per time point (except day 28 had an *n* = 4). Scale bars represent 200 *μ*m in 100× magnification, and 50 *μ*m in 400× magnification. Minor contrast and brightness adjustments were made to images to reduce background noise, but only after quantification of staining was completed.

In conjunction with cell proliferation, we also examined cellular apoptosis in the cryoinjury using TUNEL staining (Fig.7A). While apoptosis peaked in both the penumbral and direct injured areas at day 7 (Fig.7B and C), apoptosis was comparatively more prevalent in the penumbral region at earlier (day 1) and later (day 14 and 28) time points, similar to the pattern observed for cell proliferation. Furthermore, apoptosis was more extensive (by upwards of 2-fold) in the perivascular compartment, as compared to the tubular compartment.

**Figure 8:**
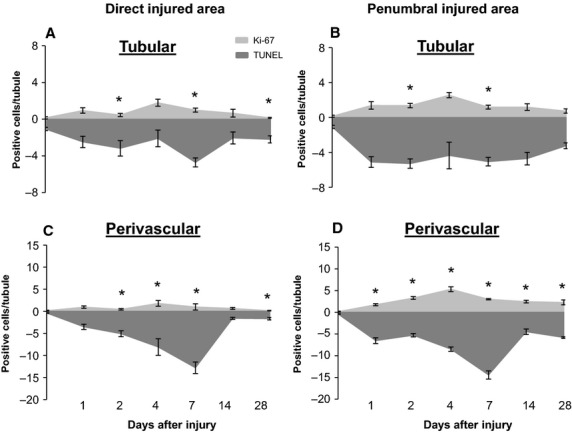
Quantified comparison of proliferating cells versus apoptotic cells at various time points after cryoinjury. The panels in the left column (A,C) represent the direct injured area, while the panels in the right column (B,D) represent the penumbral injured area. The upper panels (A,B) represent the tubular compartment, while the lower panels (C,D) represent the perivascular compartment. *The quantity of* a*poptotic cells are represented by negative numbers in each graph*. *P < 0.05 Ki-67 versus TUNEL; *n* = 5 animals per time point (except day 28 had an *n* = 4).

Comparison of tubular cell proliferation with apoptosis indicated that while a peak in proliferation was observed at 4 days postinjury in both the direct and penumbral injured areas, apoptosis remained comparably elevated at almost all time points examined with considerably more apoptosis than cellular proliferation occurring at days 2 and 7 (Fig.8A and B). In contrast, the rate of apoptosis remained prevalent over cellular proliferation in the perivascular compartments at almost all time points in both the direct and penumbral injured areas, with maximal differences observed at day 7 (Fig.8C and D).

**Figure 9:**
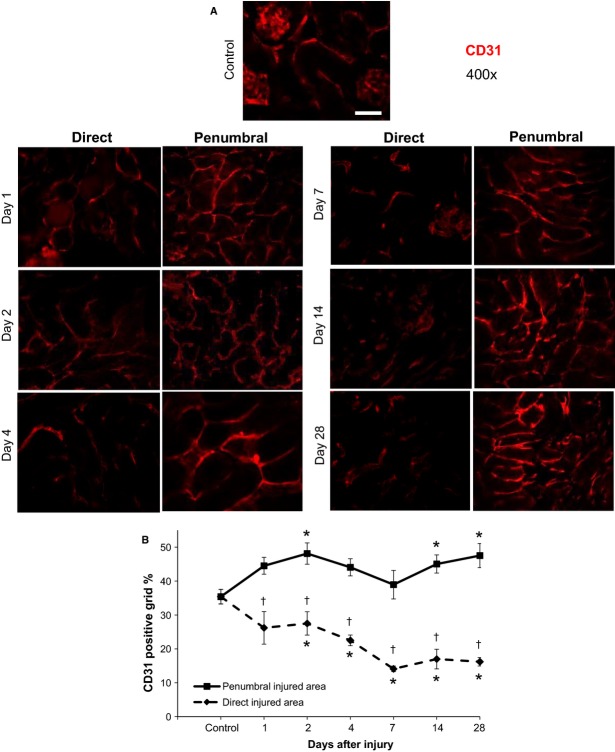
Vascular density in the kidney cryoinjury, as determined by positive CD31 immunofluorescence staining. (A) Representative images include both the direct and penumbral cryo-injured areas at 1, 2, 4, 7, 14 and 28 days postinjury. All images were taken at 400× magnification. Left panel images illustrate the direct injured area, while right panel images illustrate the penumbral injured area. Positive CD31 staining is in red. (B) CD31 positive staining in the direct and penumbral injured areas was quantified using grid analysis (explained in text) and is presented as the percentage of positive grid boxes. *P < 0.05 versus control; ^†^*P* < 0.05 direct injured area versus penumbral injured area – same day; *n* = 5 animals per time point. Scale bar represents 50 *μ*m. Minor contrast and brightness adjustments were made to images to reduce background noise, but only after quantification of staining was completed.

**Figure 10:**
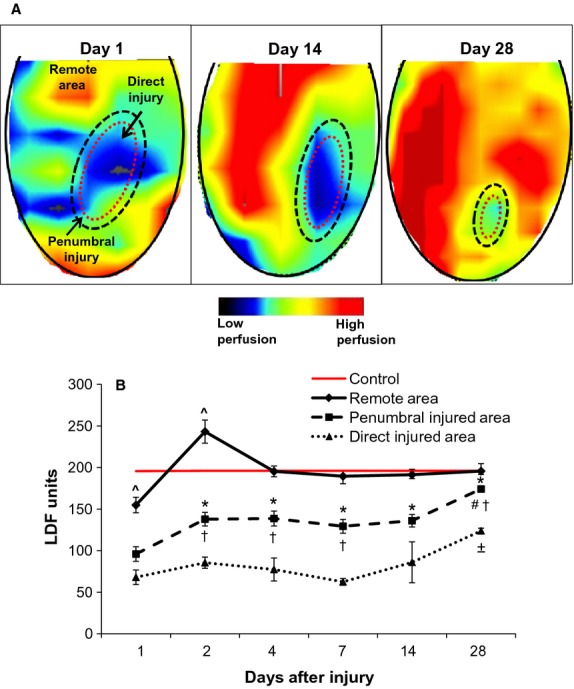
Renal blood perfusion of the cryoinjury as imaged and quantified by Laser-Doppler flowmetry. (A) Blood perfusion in the cryoinjury progressively increased during the 28 day study period, as imaged by Laser-Doppler flowmetry scanning technology. The specific areas of cryoinjury (including direct and penumbral injured areas) are outlined by the dashed ovals. (B) Blood perfusion in the direct and penumbral injured areas and also in the uninjured remote area was quantified using Laser-Doppler flow probes. Control blood perfusion was also measured in kidneys of separate healthy uninjured mice and is represented in the graph by the red line. *P < 0.05 versus penumbral injured area day 1; ^#^*P* < 0.05 penumbral injured area day 28 versus penumbral injured area days 7 and 14; ±*P* < 0.05 direct injured area day 28 versus direct injured area days 1, 2, 4 and 7; ^†^*P* < 0.05 penumbral injured area versus direct injured area – same day; ^*P* < 0.05 versus control; *n* = 6 animals per time point.

The maintained elevated incidence of apoptosis appeared to affect the vascular density within the direct injured area. Staining with the endothelial marker CD31 (Fig.9A) and subsequent quantification indicated vascular density in the direct injured area progressively declined by up 60% during the study period with the lowest levels recorded after day 4 (Fig.9B). In contrast, vascular density in the penumbral region was enhanced after cryoinjury, particularly at days 14 and 28. At all times, vascular density was 1.6- to 2.9-fold greater in the penumbral region, as compared to the direct injured area. We also examined blood perfusion (as measured by Laser-Doppler flowmetry) in the cryoinjury (Fig.10A). Since renal function was not impaired after cryoinjury, we used renal blood perfusion as a marker for evaluation of functional damage and restoration in and around the cryoinjury. On day 1 postinjury, blood perfusion was reduced by 51% in the penumbral area and 65% in the direct injured area (Fig.10B). Interestingly, in the remote area of the cryo-injured kidney, blood perfusion was also reduced by 21%, although this effect was transient and only observed within 24 h after initial injury. The remote area also subsequently underwent a surge of increased perfusion by 24% on day 2. During the 28 day study period, penumbral blood perfusion remained significantly greater (by 2-fold) than perfusion in the direct injured area, similar to results observed for CD31 in which vascular density also remained elevated in the penumbral region. At day 28, blood perfusion in the penumbral area was almost completely restored to control levels. In contrast, while blood perfusion was not completely restored in the direct injured area at day 28, probably due to the decreased vascular density that remained in this region, perfusion was re-established by 63%.

## Discussion

Data presented herein summarize our experience with renal cryoinjury and outline the protocol we have found to be optimal for kidney studies. Cryoinjury variants have been used to study repair mechanisms in the heart for nearly a century. In 1909, Eppinger and Rothberger were the first to induce a cryoinjury to heart tissue when they applied a specialized ethyl chloride spray to induce a freeze wound to a small area of the myocardium (Taylor et al. 1951). In 1920, Smith et al. induced a myocardium injury by touching the surface of the heart with a test tube filled with a hypothermic solution (Taylor et al. 1951). Methods to induce a cryoinjury continued to advance and in 1948, Nahum et al. employed a metal thermode cooled by ice water for injury induction to the epicardium and endocardium (Taylor et al. 1951). More recently, liquid nitrogen cooled cryoprobes have been developed for induction of more precise cryoinjuries. Today, cryoinjury has been examined in zebra fish (Lefer et al. 1986; Ciulla et al. 2004; van den Bos et al. 2005; van Amerongen et al. 2008; Chablais et al. 2011; Gonzalez-Rosa et al. 2011; Schnabel et al. 2011) and mammalian hearts (Laflamme and Murry 2005; Rubart and Field 2006) and has been used to unveil important aspects of heart injury and repair. For instance, the heart cryoinjury model helped confirm the discovery that resident progenitors found in the mammalian heart have the ability to differentiate into cardiac myocytes, but do not participate in the repair process (Rubart and Field 2006) leaving the injured area to be replaced by scar tissue due to prolonged inflammation (Laflamme and Murry 2005; Rubart and Field 2006). While use of the cryoinjury has evolved to allow for valuable investigation into the repair processes of the heart, the examination of a similar type of injury and potential repair in the kidney remains unexplored.

While cryoinjury in itself is not new to the kidney, its potential use as a model to study kidney damage and repair processes is. Current medical practices that target the removal of renal carcinomas utilize a specific form of kidney cryoinjury, referred to as cryoablation. Cryoablation of renal carcinomas is performed by the insertion of a cryoprobe needle into the kidney tumor, which is often located deep within the parenchymal tissue of the kidney. Once the cryoprobe is in place, argon gas circulating within the cryoprobe creates the subzero temperature which freezes the tumor. Substantial freezing of the tumor damages the cancer cells beyond repair leading to their death and eventual effective endogenous clearance. Whereas cryoablation is an effective treatment for kidney cancer, the procedure involves a long duration of cryoprobe application (typically over 10 min) in order to effectively and sufficiently freeze the cancerous lesion, a procedure which often has nonspecific deleterious effects on noncancerous kidney cells as well, potentially causing kidney damage and associated impairment of kidney function (Breining et al. 1974; Cooper et al. 1978; Sindelar et al. 1981; Barone and Rodgers 1988; Sung et al. 2003). While kidney cryoablation has been used by oncologists for the past few decades, examination of a similar but yet less severe type of cryoinjury as a model to study kidney damage and potential repair processes has not been reported. In contrast to cryoablation of carcinomas embedded within the kidney, a short transient (<1 min) cryoprobe application to the surface of the kidney induces a superficial injury characterized by a relatively modest damage that appears capable of undergoing reparative processes rather rapidly.

In our report here, we describe the postinjury events that occur after a transient kidney cryoinjury. We should first emphasize that a low scale localized kidney cryoinjury (as used in our study here) is accompanied by changes only in functional perfusion of the injury and not in overall kidney function, including no change in serum creatinine, BUN and proteinuria. After inducing a superficial cryoinjury to the mouse kidney, the area of damage underwent considerable apoptosis and cell proliferation, both of which peaked by the end of the first week following injury, at which time macrophage infiltration was also elevated. H&E analysis indicated the cryo-induced lesion expanded within the first few days after initial insult, but then substantially decreased in size by 73% by the end of the 28 day study period. Similarly, we also saw a progressive increase in fibrosis within the first 7 days of injury. After day 7, fibrosis began to diminish.

The initial cryoinjury lesion expansion that occurred at day 7 (indicated by H&E and trichrome staining in Fig.2A) and the expansion of fibrosis into deeper cortical and medullary regions at days 4, 7 14 and 28 (Fig.3) may be attributed to multiple factors. As observed by CD31 staining (Fig.9) and renal blood perfusion measurement (Fig.10), the cryoinjury was characterized by vascular damage in and around the lesion. The descending capillary network that emerges from cortical efferent arterioles is responsible for supplying nephron segments that are deeper in the cortex and medulla with crucial blood and oxygen that is critically required to maintain normal cellular processes, ATP generation and tubular solute transport (Eckardt et al. 2005; Leong et al. 2007). Thus, for adequate blood and oxygen delivery to these nephron segments deeper in the kidney, adequate upstream cortical blood perfusion is necessary (Eckardt et al. 2005; Leong et al. 2007). While the kidney is sensitive to ischemic injury (Brezis and Rosen 1995; Pallone et al. 2003), the corticomedullary and medullary aspects of the nephron are especially sensitive to decreased oxygen supply (Pallone et al. 1990, 1998; Whitehouse et al. 2006; Joo et al. 2007; Legrand et al. 2008), which results in cellular stress, generation of reactive oxygen species, inflammation and fibrosis in these areas. In addition, the tissue within the direct cryo-injured area would expectedly release various alarmins, such as HMGB1, which promote local and systemic pro-inflammatory processes (Ratliff et al. 2013). These signaling molecules diffuse to adjacent cortical and medullary tissues via the microvascular circulation, tubular fluid and interstitial fluid, and activate chemotactic and cytokine proinflammatory processes. These processes subsequently propagate the damage to areas adjacent to the direct (cryo-)injured area, including distal cortical and medullary regions. However, the diffused potentiated damage begins to recede once reparative processes begin.

While exact boundaries of the penumbral region could not be precisely demarcated, the penumbral zone was clearly distinguishable by blood perfusion, immunofluorescence and immunohistochemistry staining patterns despite some relative blurring of the borders between the penumbral area and either the direct or healthy regions. It is expected that the minor inaccuracies that arise due to the inability to exactly define borders are negligible due to the overall significant differences observed deep within the penumbral zone, particularly since the assessment of the dynamics of this zone is based heavily on events that occur centrally in the penumbral zone instead of solely on border events. As compared to the direct injured area, we observed earlier macrophage infiltration in the penumbral injured area, along with more robust rates of cellular proliferation and apoptosis, suggestive of rapid cell turnover. Vascular differences between the two regions were also more pronounced. The penumbral region had preserved vascular density after injury, while the direct injured area did not. Despite an overall reduction in blood perfusion after injury, the penumbral area demonstrated enhanced perfusion, as compared to the direct injured area, with almost complete restoration of perfusion at day 28. The increase in vascular density and blood perfusion of the penumbral injured area would be expected to help with reparative processes in and around the most severely damaged tissue by supplying these areas with critically needed metabolic and cellular requirements. Furthermore, the observed reduction in the size of the cryo-induced lesion along with improved blood perfusion of the injury during the 28 day study period suggests the renal extracellular matrix and structural scaffolding remain intact after cryoinjury allowing for reparative processes to occur.

While we also report on differences in tubular and perivascular damage and reparative events in the cryoinjury, which includes enhanced apoptosis and macrophage infiltration in the perivascular compartment, we refrain from reporting on glomerular damage and repair dynamics. Since the cryoinjury was confined to the outer rim of the cortex, we did not consistently observe enough glomeruli in the samples to adequately characterize the effect of cryoinjury on these structures. However, increasing the duration of the initial cryoprobe induced insult to the kidney would presumably result in a more robust injured area, which should allow the inclusion of a greater number of glomeruli. This also highlights an additional advantage of using the cryoinjury model, its flexibility. A cryoinjury can be induced anywhere and of variable severity.

The use of the cryoinjury model provides various advantages for the study of kidney damage and repair mechanisms, as compared to other currently used kidney injury models. A significant advantage of the cryoinjury model is that injury induction is simple and rapid with virtually no complications. The injury can be induced bilaterally or unilaterally to kidneys through a minimally invasive midlaparotomy or flank retroperitoneal incision. Animals tolerate the procedure and cryoinjury remarkably well and spend a minimal amount of time under anesthesia. In fact, we had a zero mortality rate of mice that received the cryoinjury. The extremely low rate of mortality is a tremendous asset to this model. Furthermore, the ease of application permits a vast number of animals to receive the cryoinjury in a short period of time allowing for sufficient sample sizes and biological replicates to be obtained quickly and numerous time points to be examined. Induction of a cryoinjury is inexpensive and since the animals tolerate well the surgery and injury, animal mortality does not enhance the experimental costs.

To provide more reproducible results using renal ischemia-reperfusion injury models, the ischemic period needs to be expanded to over 45 min and introduced bilaterally or instead include unilateral nephrectomy (Skrypnyk et al. 2013). However, at longer ischemic times, many of the animals die within the first few hours to within days after injury, thus impairing the ability to fully study reparative processes unless numerous animals are used at a large financial cost. Sustained kidney injury is variable in other models as well. For instance, introduction of toxins including lipopolysaccharide or bacteria at low to mid-range doses often does not consistently induce organ failure, induces an inconsistent kidney injury, or induces a mildly transient kidney injury, while higher doses often dramatically enhance mortality (Heyman et al. 1996, 2000). Pigment nephropathy models are also used and provide reliable reproducible results, but often these models induce a kidney injury too severe (as with glycerol injection) (Zager 1996) or too mild (myoglobin or hemoglobin infusion) (Heyman et al. 1996). While contrast nephropathy models are clinically relevant, this models is highly complex with large variability between animals (Heyman et al. 1999). Other models of kidney injury do not induce a global injury that affects both vascular and tubular structures. In contrast, while some variability is observed between animals that receive a cryoinjury, the variability is minimal and can be effectively overcome by standardization of techniques used to induce the initial cryoinjury (as described here in the methods section). The degree of kidney damage sustained during cryoinjury affects both tubular and vascular structures and can be induced at a variable intensity, a significant benefit of this model. In mildly induced cryoinjuries (as used in our studies here), damage is not severe enough to impeded reparative processes as crucial underlying regenerative signaling, structural integrity and matrix components of the kidney architecture are preserved. Furthermore, the size of cryoinjury is localized to one area of the kidney instead of diffused throughout the entire kidney, allowing for complete examination of the injured area, surrounding tissue, and the interaction of these two zones. This characteristic of the cryoinjury allows the examination of the reparative dynamics associated with the penumbral zone and its effect on the direct injured area, including repair and shrinkage of the injury lesion. In most other kidney injury models, this dynamic is unobservable due to the widespread nature of the injury and the lack of an identifiable penumbral transitional zone.

In conclusion, the study offers insight into the superficial mouse kidney cryoinjury model including its potential usefulness for studying kidney damage and/or reparative mechanisms.

## Conflict of Interest

All authors declare no competing interests.
